# A Qualitative Study of “What Matters” to Older Adults in the Emergency Department

**DOI:** 10.5811/westjem.2022.4.56115

**Published:** 2022-07-01

**Authors:** Cameron J. Gettel, Arjun K. Venkatesh, Hollie Dowd, Ula Hwang, Rockman F. Ferrigno, Eleanor A. Reid, Mary E. Tinetti

**Affiliations:** * Yale School of Medicine, Department of Emergency Medicine, New Haven, Connecticut; † Yale School of Medicine, Center for Outcomes Research and Evaluation, New Haven, Connecticut; ‡ Yale School of Public Health, New Haven, Connecticut; § Yale New Haven Health – Bridgeport Hospital, Department of Emergency Medicine, Bridgeport, Connecticut; ¶ Yale School of Medicine, Department of Internal Medicine, New Haven, Connecticut; || Yale School of Public Health, Department of Chronic Disease Epidemiology, New Haven, Connecticut

## Abstract

**Introduction:**

The “4Ms” model – What Matters, Medication, Mentation, and Mobility – is increasingly gaining attention in age-friendly health systems, yet a feasible approach to identifying what matters to older adults in the emergency department (ED) is lacking. Adapting the “What Matters” questions to the ED setting, we sought to describe the concerns and desired outcomes of both older adult patients seeking ED care and their treating clinicians.

**Methods:**

We conducted 46 dyadic semi-structured interviews of cognitively intact older adults and their treating clinicians. We used the “What Matters” conversation guide to explore patients’ 1) concerns and 2) desired outcomes. We then asked analogous questions to each patient’s treating clinician regarding the patient’s priorities. Interviews were professionally transcribed and coded using an inductive approach of thematic analysis to identify emergent themes.

**Results:**

Interviews with older adults lasted a mean of three minutes, with a range of 1–8 minutes. Regarding patients’ concerns, five themes emerged from older adults: 1) concern through a family member or outpatient clinician recommendation; 2) no concern, with a high degree of trust in the healthcare system; 3) concerns regarding symptom cause identification; 4) concerns regarding symptom resolution; and 5) concerns regarding preservation of their current status. Regarding desired outcomes, five priority themes emerged among older adults: 1) obtaining a diagnosis; 2) returning to their home environment; 3) reducing or resolving symptoms; 4) maintaining self-care and independence; and 5) gaining reassurance. Responding to what they believed mattered most to older adult patients, ED clinicians believed that older adults were concerned primarily about symptom cause identification and resolution and primarily desired a return to the home environment and symptom reduction.

**Conclusion:**

This work identifies concerns and desired outcomes of both older adult patients seeking ED care and their treating clinicians as well as the feasibility of incorporating the “What Matters” questions within ED clinical practice.

## INTRODUCTION

Older adults (those aged 65 years and over) account for over 23 million emergency department (ED) visits annually, representing 18% of all ED visits nationally.^1^ Older adults have been noted to face unique challenges related to emergency care, including the potential receipt of goal-discordant care and a decreased attention to patient-centered care.^2^,^3^ As a potential solution to address the underlying problems facing older adults more broadly in healthcare settings, the John A. Hartford Foundation and the Institute for Healthcare Improvement (IHI) founded the Age-Friendly Health Systems initiative in 2017.^4^–^7^ As of June 2021,^4^ there were over 2200 age-friendly health system participants employing the framework called the “4Ms” – What Matters, Medication, Mentation, and Mobility – to ensure patient-centered and evidence-based care for older adults across healthcare settings, with wider implementation in ambulatory and inpatient settings and less attention to the ED setting.^8^–^10^

Within EDs, efforts are increasing to prioritize patient-centeredness and goal-concordant care for older adults.^11^,^12^ These areas of focus are particularly relevant and important for older adults as they have been identified in the outpatient setting to have health-related priorities aside from typical metrics such as repeat ED visits or hospitalization.^13^–^15^ Furthermore, much of the available emergency care research regarding older adults’ patient-centered goals currently focuses on treating clinicians performing end-of-life goals of care conversations.^16^–^18^ However, ED treating clinicians are tasked with navigating older adult priorities not just during critical illness or end of life.

To date, the extant literature has not assessed whether ED treating clinicians perceive priorities that differ from their older adult patients, thereby potentially introducing goal-discordant care. Identifying what matters and priorities within the broader older adult population has drawn concerns regarding implementation strategies in the ED clinical environment as the lines of questioning often are perceived as time-intensive^19^ or beyond the scope of a traditional ED visit focused on a single injury or symptom. Thus far, a feasible approach aligned with the Age-Friendly Health Systems to identify what matters to older adults seeking emergency care is lacking. Therefore, we sought to describe the priorities identified by older adults’ and their treating clinicians as well as the feasibility of incorporating brief questions addressing what matters in the ED. Identifying the concerns and desired outcomes of older adults in a time-efficient approach that is aligned with the “What Matters” domain of the 4Ms framework will allow more patient-centered ED care for this growing population.

## METHODS

### Study Design

We performed a qualitative analysis involving cognitively intact patients and their treating clinicians. Study methods and results are presented in accordance with the consolidated criteria for reporting qualitative research (COREQ).^20^ This study was determined to be exempt research by the institutional review board.

*Population Health Research Capsule*

What do we already know about this issue?

*Older adults face unique challenges related to emergency care, including decreased attention to patient-centered and goal-concordant care.*

What was the research question?

*Can emergency clinicians identify concerns and desired outcomes of older adults using the “What Matters” conversation guide?*

What was the major finding of the study?

*“What Matters” questions in the ED are feasible, with clinicians and older adults exhibiting varied alignment.*

How does this improve population health?

*Identifying what matters to older adults should spur emergency physicians to pursue an evaluation, treatment plan, and disposition aligned with patients’ goals.*

### Sample

The study was conducted at two EDs – a community hospital and a Level II trauma center – within the same health system. Potential older adult participants were identified based on screening within the electronic health record, with recruitment taking place during rotating evening and day schedules. Inclusion criteria included the following: age **≥**70; English-speaking; ability to answer questions without the assistance of caregivers; and an Emergency Severity Index score of 3, 4, or 5 suggesting lower acuity at triage. Exclusion criteria included a status of medically unfit (as determined by the treating clinician) or evidence of cognitive impairment. We used the six-item screener, with a score of <4 on the six-point questionnaire indicating high risk for cognitive impairment, as previously performed in ED-based research.^21^ Treating clinicians, including attending physicians and non-physician practitioners (eg, physician assistant, nurse practitioner), received a $5 gift card for their time participating in the interview. Enrollment occurred between December 2020–May 2021.

### Procedures

A trained interviewer (HD) obtained verbal consent and digitally recorded interviews of older adults and their treating clinicians. We conducted semi-structured interviews with a sample of older adult ED patients using an interview framework, the “What Matters in the ED” conversation guide (Supplement 1). The guide was modified from another Patient Priorities Care guide and developed by stakeholders and experts in work related to age-friendly health systems and emergency care.^22^ Contextually, the “What Matters” conversation guide was developed to align the IHI Age-Friendly Health System initiative with the Geriatric ED Accreditation process endorsed by the American College of Emergency Physicians. The purpose of the “What Matters” conversation guide was to provide an outline for emergency clinicians to ask and learn about what matters to older adults presenting to the ED, with the knowledge gained contributing to care and treatment decisions.

An initial version of the “What Matters” conversation guide was tested in three EDs to gain clinician insights regarding appropriateness and feasibility (Supplement 1). We used the final two questions previously identified by expert consensus deemed to be most salient to identify what matters for older adults seeking emergency care.^23^ To assess concurrent clinician impressions of their older adult patients, we asked analogous questions in a separate interview to the patient’s ED treating clinician regarding what they believed mattered most to the older adult they were treating (Table 1). As suggested by stakeholder and expert guidance on the “What Matters” conversation guide, HD could ask either question 1a or 1b to ascertain fears or concerns about the older adult’s healthcare in the ED, with a similar approach suggested for question 2a or 2b to identify outcomes most wanted. When identifying fears or concerns, HD’s approach was to start the interview by asking question 1a. HD asked question 1b if the participant had difficulty understanding the question, needed further clarification, or it was thought that greater information could be gathered by rephrasing the question. The final interview guide was pilot tested with two ED patients prior to beginning the study.

Both patients and clinicians were interviewed during the ED encounter when disposition uncertainty still existed. This occurred after the initial evaluation by the treating clinician, but before laboratory and imaging results were available to inform decision-making. HD timed interviews from the start of asking question 1 to the end of the participant’s response to question 2 to assess the time and operational feasibility of incorporating “What Matters” questions into a typical ED encounter. HD collected basic demographic information and ED clinical data regarding the encounter and also recorded brief field notes immediately after the interview. No study authors were part of the participants’ medical care teams.

### Data Analysis

We used an iterative process of thematic analysis to synthesize the data, identify patterns, and develop themes across interviews.^24^ Specifically, we used the inductive qualitative approach that relies on the synthesis of qualitative data, rather than relying on concepts considered a priori.^25^ The coding team consisted of CJG, an emergency physician and health services researcher with formal qualitative training and expertise working with older adults, and HD, a masters-level research associate whom CJG trained on qualitative research techniques. Digitally recorded transcripts were professionally transcribed and corrected when the transcript passage was incomprehensible or had errors. We used NVivo 12 qualitative software (QSR International, Melbourne, Australia) to manage and analyze study data.^26^

The coding team began with a line-by-line review of transcripts and open coding to identify key concepts. Following review of the first six transcripts, coders developed an initial codebook that was subsequently expanded and refined through independent and then joint review of additional transcripts. Coding discrepancies were adjudicated between coders through regular meetings, and the final codebook, containing 22 codes across four domains, was then applied to all transcripts. Both coders coded all interviews to enhance consistency. Recruitment, interviewing, and coding occurred concurrently until thematic saturation was reached.^27^ We followed best practices for validity in qualitative research by maintaining an audit trail and comments and revisions from group coding meetings.^28^–^30^ The study team collaboratively identified and agreed upon illustrative quotes the represented the identified final themes. To preserve anonymity, participant quotes are identified by participant type and number.

## RESULTS

We screened 58 older adults for eligibility; eight refused to participate and four were noted to be cognitively impaired, leaving 46 older adults and their treating clinicians who agreed to participate and completed interviews. Older adult participants were primarily female, White, married, and had a mean age of 87 years. Characteristics of participants are shown in Table 2. The most common ED chief complaint category was “fall, musculoskeletal,” and a significant portion of older adults underwent both laboratory testing (93%) and radiograph imaging (70%). Interviews with older adults lasted a mean of three minutes, with a range of 1–8 minutes. Treating clinicians consisted of physicians and non-physician practitioners (Table 2).

When considering responses to the first “What Matters” question regarding fears or concerns about the older adult’s ED care, five main themes emerged among older adult respondents and two main themes emerged among clinician respondents. For older adults, these themes included the following: 1) concern through a family member or outpatient clinician recommendation; 2) no concern, with a high degree of trust in the healthcare system; 3) concerns regarding symptom cause identification; 4) concerns regarding symptom resolution; and 5) concerns regarding preservation of their current status. For clinicians responding to what they believed the older adult patient was most concerned about, the two themes included 1) concerns regarding symptom cause identification and 2) concerns regarding symptom resolution.

When considering responses to the second “What Matters” question regarding desired outcomes about the older adult’s ED care, five main themes emerged among older adult respondents and three main themes emerged among clinician respondents. For older adults, these themes included the following: 1) obtaining a diagnosis; 2) returning to their home environment; 3) reducing or resolving symptoms; 4) maintaining self-care and independence; and 5) gaining reassurance. For clinicians responding to what outcomes they believed the older adult patient most desired, the three identified priority themes included 1) returning to their home environment, 2) linking reassurance and return to home environment outcomes, and 3) reducing or resolving symptoms. Tables 3 and 4 show representative quotes of the identified themes.

### Insights into Older Adults Concerns

Older adults reported a wide variation of concerns when thinking about their health and healthcare during the ED visit. Older adults either presented to the ED at the suggestion of a family member or the recommendation of an outpatient clinician, while an additional group were not concerned at all with their ED care and noted their “total confidence” in the ED treating clinicians. When present, concerns and fears of older adults included symptom cause identification and symptom resolution (eg, knee pain), but more frequently also extended to include the ramifications that the acute injury or illness would have on their broader life. These areas of concern for older adults centered commonly on ambulatory status and preservation of their current abilities (Table 3). One participant stated,*”I am just concerned that I will not get back to normal.” (Participant)*

When asked to consider the older adults’ concerns, treating clinicians referenced symptom cause identification and symptom resolution as the patient’s greatest concern or fear, with no comment on the perceived impact that the older adult identified on daily life or function. Highlighting potential discordance regarding concerns, one older adult and their treating clinician separately noted:

*“I have a heart condition. I have an artificial knee, and it looks like I might be getting another artificial knee. Mobility is the big issue.” (Participant)*

*“I think he is concerned about his left knee pain that is recurrent.” (Clinician)*

### Insights into Older Adults Desired Outcomes

Older adults and clinicians also reported a wide array of desired outcomes for the older adult during the ED visit. Individuals from both groups identified that the desired outcomes of older adults during ED care included returning to their home environment and reducing or resolving symptoms. Highlighting concordance between patients and treating ED clinicians, one older adult and their treating clinician separately noted:

*Interviewer: “What outcomes are you most hoping for from this ED visit?”*

*Participant: “To be able to go back to the facility where I reside.” (Participant)*

*“I think ultimately she would like to be discharged and be told everything is looking good.” (Clinician)*

Expressing desired outcomes from their ED visit, an older adult and their treating clinician also noted:

*“That my head is clear, and I can go home and get on with my life.” (Participant)*

*“I think they would like to go home, but they are also concerned about his head.” (Clinician)*

However, older adults additionally noted obtaining a diagnosis, maintaining self-care and independence, and gaining reassurance as desired outcomes from their ED visit. Treating clinicians linked desired outcomes of older adults, most commonly identifying their desire to gain reassurance alongside their desire to return to their home environment. However, clinicians did not perceive that maintaining self-care and independence were desired outcomes of older adults seeking emergency care (Table 4). Highlighting potential discordance regarding desired outcomes, one older adult and their treating clinician separately noted:

*“I want to get back to where I can be myself because I used to love to exercise. I used to love to walk, and it seems like I can’t do any of that now. I’m an independent person, and I like doing for myself, and I hate when I have to have other people do for me.” (Participant)*

*“I think they want an answer as to the cause of the shortness of breath primarily, and then also to treat it.” (Clinician)*

## DISCUSSION

This study is the first to characterize perspectives of older adults presenting to the ED using the “What Matters” framework. The unique comparison to their treating clinicians offers evidence demonstrating alignment in some areas despite other distinct gaps between older adults and their ED treating clinicians. Importantly, this work identifies the feasibility of incorporating the “What Matters” questions in the ED.

Unique to our work is the identification of what older adults are concerned about and prioritize while seeking emergency care, *and* whether clinicians are aware of what matters to this population. In our study, clinicians often recognized the importance of returning to the home environment for older adults, but they did not comment on patients’ frequently expressed concerns regarding the impact of the acute illness or injury on their ability to return to their previous functional or broader health status. The emergency clinicians rarely mentioned functional changes as a concern of the older adult despite prior ED- and hospital-based literature identifying subsequent objective functional decline and adverse outcomes.^31^–^36^ Our qualitative study adds to the literature base by providing more in-depth responses than possible via survey-based quantitative research.

The extant literature lacks relevant feasible modalities to address the priorities of older adults seeking emergency care. Many, including the “What Matters” structured tool and the “Serious Illness Conversation Guide,^37^ have been developed and assessed in non-ED settings, thereby limiting their translatability to patients seeking acute care. In our study, the average patient interview was three minutes and ranged from 1–8 minutes, suggesting a reasonable time to completion and feasibility of clinicians incorporating the “What Matters” questions within the time constraints of today’s ED clinical practice. We believe emergency clinicians are best situated to ask the “What Matters” questions, as their upfront efforts to address patient priorities, concerns, and desired outcomes may ultimately save time and resources in place of potentially contentious and goal-discordant conversations after completion of the ED evaluation.

Additionally, Hunold et al asked a single, open-ended question to older adults regarding what would make their ED visit successful, useful, or valuable.^38^ Without restricting when during the visit the interview occurred, 62% of participants reported at least one priority in the “evaluation, treatment, and outcomes” meta-category, including treatment of the medical problem, accurate diagnosis, and competent clinical staff. Our study builds upon this work by providing more in-depth qualitative responses as well as standardizing the interview time during the clinical encounter – after initial clinician evaluation but before laboratory and imaging results. This timing ensured uncertainty regarding the disposition and allowed patients to reliably and consistently relay priorities at a critical juncture in the ED visit. It remains to be determined whether these questions may be most effective in guiding ED care if asked at the initiation of the visit, prior to evaluation.

Our work has several implications regarding clinical practice. Cognitively intact older adults identified several concerns regarding their health in comparison to their treating clinicians, suggesting that emergency clinicians may be unaware of certain patient priorities during the encounter. A standardized script, such as the “What Matters” conversation guide, may prompt clinicians to incorporate patient-centeredness and shared-decision making into the patient encounters. Identifying what matters in the ED when caring for older adults is intended to promote clinicians to pursue an evaluation, treatment plan, and disposition aligned with the goals of the patient, potentially saving both time and financial resources if an extensive in-ED evaluation is not prioritized or desired by the patient. The Age-Friendly Health System initiative may serve as a platform for the broader implementation in the ED of the “What Matters” conversation guide to target increased patient-centered emergency care of older adults, creating alignment with the recent development of geriatric ED guidelines and the Geriatric ED Accreditation (GEDA) process.^39^,^40^ Future research should build upon this foundation and quantitatively identify domains targeting what matters that can be incorporated within novel patient-reported outcome measures and may benefit from determining differences between GEDA and non-GEDA EDs in addressing the “What Matters” questions.

## LIMITATIONS

There are limitations of our study to consider. Our study was conducted at EDs within one health system and predominantly among White older adults, thereby potentially restricting generalizability. However, we expect that many older adults will have similar experiences as we identified thematic saturation during our qualitative analyses. Our understanding and interpretation of the data may have potentially introduced confirmation bias, which we attempted to minimize using semi-structured interview guides and discrepancy reconciliation through team discussion. Additionally, the two primary interview reviewers were not blinded to the study objectives, thereby potentially introducing bias to the decision of classification of the questionnaire domains. While we did follow multiple best practices for rigor in qualitative research,^28^–^30^ we did not return transcripts to participants for checking of our themes. Finally, “feasibility” has been defined in several ways within the literature. Aside from the time taken to conduct the interviews, additional quantitative survey feedback from older adults and ED treating clinicians may be beneficial to support further operational implementation.

## CONCLUSION

Patients and their treating clinicians noted similar concerns and desired outcomes when considering the priorities of older adults. However, clinicians did not as frequently recognize patients’ concerns about the impact of their acute condition on overall function and daily life. We have identified the feasibility of incorporating these two “What Matters” questions in the ED and the limited time needed to identify older adults’ priorities

## Supplementary Information



## Figures and Tables

**Table 1:** ”‘What Matters” semi-structured interview guide for older adult patients and their treating clinicians.

Questions for older adult patients One question to ascertain fears or concerns about healthcare in ED What concerns you most when you think about your health and about being in the ED today/tonight? or What fears and worries do you have about your health as you think about what brought you to the ED today/tonight? One question about outcome patients most want from their ED visit What outcome are you most hoping for from this ED visit? or What are you most hoping for or looking for from your ED visit?
Questions for treating clinicians What do you think the patient/family is concerned about today? What outcomes do you think the patient is most hoping for?

*ED*, emergency department.

**Table 2:** Sample characteristics.

Variable	N = 46
Age, mean (SD)	87 (7)
Female, n (%)	27 (57)
Race, n (%)	
White	37 (80)
Black	7 (16)
Other	2 (4)
Ethnicity, n (%)	
Hispanic or Latino	2 (4)
Non-Hispanic	44 (96)
Marital Status, n (%)	
Single	4 (9)
Married	22 (48)
Divorced	3 (6)
Widowed	15 (33)
Other	2 (4)
Chief Complaint Category, n (%)	
Fall, musculoskeletal	16 (35)
Weakness, fatigue, dizziness	11 (24)
Cardiopulmonary	10 (22)
Other	9 (19)
ED Evaluation, n (%)	
Labs	43 (93)
Radiograph	32 (70)
Ultrasound	4 (9)
CT imaging	16 (35)
Final ED Disposition, n (%)	
Admit	26 (57)
Discharge	20 (43)
ED Diagnosis Category, n (%)	
Musculoskeletal	12 (26)
Infection	7 (15)
Cardiopulmonary	8 (18)
Metabolic, electrolyte disturbance	7 (15)
Other	12 (26)
Interview time of day, n (%)	
9 AM–4 PM	16 (35)
4 PM–11 PM	30 (65)
Clinician type, n (%)	
Physician	34 (74)
Non-physician practitioner	12 (26)
Average patient interview length, min (range)	3 (1–8)

*SD*, standard deviation; *ED*, emergency department, *CT*, computed tomography.

**Table 3:** Fears and concerns related to emergency care of older adults and their treating clinicians.

Question #1 – Fears and concerns about healthcare in the ED?	

Theme	Exemplar quotes
*Older adults*	
**Theme 1:** Concern through a family member or outpatient clinician recommendation	One of my doctors did not like the result of a blood test that I had taken last Friday and he did not like the result of my blood pressure today. (Participant)
	I really did not want to come, but my kids made me come. (Participant)
	Well, I haven’t paid a great deal of attention to my health. My wife has been at me to be more concerned about how I feel, what’s happening, and what I need to feel better. (Participant)
**Theme 2:** No concern, with a high degree of trust in the healthcare system	When I came into the emergency department tonight, I was treated with respect, and I love being here because at least I get some progress. (Participant)
	I don’t know if I have any concerns, because I have total confidence that they are going to take care of the problem. (Participant)
	Nothing really, because I’m in good hands. They know what they’re doing. I have no worries about it. (Participant)
**Theme 3:** Concerns regarding symptom cause identification	I would say what concerns me the most is finding out what is my problem. (Participant)
	What concerns me most is that I just want to find out what’s going on with my health and my body. (Participant)
	Finding out what is really wrong with me. (Participant)
	Finding out what is wrong with me. (Participant)
**Theme 4:** Concerns regarding symptom resolution	I have pain in my left hip, which is totally unexplainable. (Participant)
	Being able to feel better and poop, because I have been eating, but nothing’s been coming out and it’s very uncomfortable. (Participant)
	Getting rid of the pain that I got. (Participant)
	To get better…I felt like I was going to faint and my legs gave way. (Participant)
	I feel awful, I feel so nauseous. So that is concerning to me be because I really do not want to mess myself or anything you know. (Participant)
**Theme 5:** Concerns regarding preservation of their current status	Mobility – I have a heart condition, an artificial knee – mobility is the big issue. (Participant)
	I am just concerned that I will not get back to normal. (Participant)
	I fell, and if my knee gets hurt I don’t know if I will ever walk again. (Participant)
	I want to be by myself, and I want to take care of myself. I don’t move much at home because I have a hard time getting up and moving. (Participant)
*Clinicians*	
**Theme 1:** Concerns regarding symptom cause identification	I think he is most concerned about the source of his pain. (Clinician)
	He is definitely concerned about his left knee pain. He thinks he has another infection, because he has a history of similar. (Clinician)
	I think one of the main things that he is concerned about is the dizziness that he does not know where it is coming from. (Clinician)
**Theme 2:** Concerns regarding symptom resolution	I think he is concerned that he has an infection that has not been improving on antibiotics. (Clinician)
	Persistent shortness of breath that has not been treated. (Clinician)
	The pain in her back. (Clinician)
	He was concerned that he was not urinating. (Clinician)
	The patient’s main concern was the discomfort in her right shoulder and left knee after falling today. (Clinician)

**Table 4:** Desired outcomes related to emergency care of older adults and their treating clinicians.

Question #2 – Outcome most hoping for from this ED visit?	

Theme	Exemplar quotes
*Older adults*	
**Theme 1:** Obtaining a diagnosis	The doctors will find whatever is causing the pain and we will just move on. (Participant)
	To actually just learn what is wrong. (Participant)
	Find out what is causing this. (Participant)
**Theme 2:** Returning to their home environment	That I do not have to have any operations and I can go home soon. (Participant)
	To go home. (Participant)
	Nothing really, because I’m in good hands. They know what they’re doing. I have no worries about it. (Participant)
	That I can be bandaged up and go home. (Participant)
	Recovery back home. (Participant)
	To be able to go back to the facility where I reside. (Participant)
**Theme 3:** Reducing or resolving symptoms	No recurring symptoms. (Participan)
	Get rid of the pain. I can tolerate discomfort, but pain management today. (Participant)
	I am hoping that my stomach will go down and I will [be] able to poop and feel better. (Participant)
	To get rid of the pain. (Participant)
**Theme 4:** Maintaining self-care and independence	I want to get back to where I can be myself because I used to love to exercise. I used to love to walk and it seems like I can’t even do none of that now, and I am an independent person and I like doing for myself and I hate when I have to have other people to do for me. (Participant)
	That I know what to do to better take care of myself. (Participant)
**Theme 5:** Gaining reassurance	Something that doesn’t incur surgery. (Participant)
	Everything is normal. (Participant)
	I hope there is nothing wrong. (Participant)
*Clinicians*	She wants to go home. (Clinician)
**Theme 1:** Returning to their home environment	Could be able to be discharged home. I think it is what she is hoping for. (Clinician)
	Ability to go back to Assisted Living. (Clinician)
	I think he hoped that he could go home. (Clinician)
**Theme 2:** Linking reassurance and return to home environment outcomes	He is hoping that I tell him that that is not the case [an infected knee] and he gets to go home. (Clinician)
	I think that she is hoping that everything is negative and she gets to go home. (Clinician)
	To be discharged from the emergency department today, and to have reassurance that he does not have a fracture or new blood clot. (Clinician)
	I think she was hoping that she would be cleared with basic emergency department evaluation and be able to go home. (Clinician)
	I think ultimately she would like to be discharged home and be told everything is looking good. (Clinician)
**Theme 3:** Reducing or resolving symptoms	The bleeding to stop. (Clinician)
	Probably pain control and her arm healing. (Clinician)
	To feel better and not be short of breath. (Clinician)

*ED*, emergency department.
