# Supplementary material for: A Qualitative Study of “What Matters” to Older Adults in the Emergency Department
Source: West J Emerg Med. 2022 Jul 1;23(4):579–88. doi: 10.5811/westjem.2022.4.56115 (PMC9391017; doi:10.5811/westjem.2022.4.56115)
Supplement: Supplementary file 1 [file wjem-23-579-s001.docx]

# ‘What Matters’ in the ED: Conversation Guide

| **Clinician Steps** | **What: Suggested Wording** | **Rationale** |
| --- | --- | --- |
| 1. Let patients know why you are asking these questions. | *“In our ED, we want to understand what matters to you about health and your healthcare, to make sure that the care we give during your time in the ED, as well as after discharge, is right for you.”* | People may not expect these questions, so this sentence is meant to explain/provide context. |
| 2. One question to ascertain concerns and fears about health and healthcare in the ED. | Option 1: *“What concerns you most when you think about your health and about being in the ED today/tonight?”*  Option 2: “*What fears and worries do you have about your health as you think about what brought you to the ED today/tonight?”* | Giving the patient an opportunity to share his/her fears and concerns about his/her health and about being in the ED helps you tailor treatment and education, with the aim to increase the effectiveness and efficiency of ED care. |
| 3. One question about the outcome patients most wants from their ED visit. | *“What outcome are you most hoping for from this ED visit?”* | To align care with what matters most to each person, help the patient identify a desired outcome the patient hopes to achieve. |

Dr. Mary Tinetti and Dr. Kevin Biese 17 May 2019

**Purpose**: To provide an outline for ED care providers to ask and learn about What Matters to older patients who come to the ED^[[1]](#footnote-1)^. Knowledge of what matters should contribute to effectiveness and efficiency of care.

**Context:** We have identified opportunity to align IHI Age Friendly Health Systems recognition with the ACEP Geriatric ED Accreditation program. The What Matters Conversation Guide outlined here is part of that alignment.

1. e.g. patients at levels 3, 4 and 5 in a five-level triage severity index system; such patients typically represent more than 50% of patients seen in the ED. [↑](#footnote-ref-1)
